# An Assessment of Childbearing Preferences in Northern Malawi

**DOI:** 10.1111/j.1728-4465.2015.00022.x

**Published:** 2015-06

**Authors:** Kazuyo Machiyama, Angela Baschieri, Albert Dube, Amelia C. Crampin, Judith R. Glynn, Neil French, John Cleland

**Affiliations:** Faculty of Epidemiology and Population Health, London School of Hygiene and Tropical Medicine, Keppel Street, London, WC1E 7HT, United Kingdom; University of Southampton; Community Health Department, College of Medicine, University of Malawi; Faculty of Epidemiology and Population Health, London School of Hygiene and Tropical Medicine, Keppel Street, London, WC1E 7HT, United Kingdom; Faculty of Epidemiology and Population Health, London School of Hygiene and Tropical Medicine, Keppel Street, London, WC1E 7HT, United Kingdom; Institute of Infection and Global Health, University of Liverpool; Faculty of Epidemiology and Population Health, London School of Hygiene and Tropical Medicine, Keppel Street, London, WC1E 7HT, United Kingdom

## Abstract

Fertility preferences are an essential component of family planning program evaluation; however, doubts about their validity in sub-Saharan Africa exist and little methodological assessment has been carried out. This study investigates prospective fertility intentions in terms of their temporal stability, intensity, degree of spousal agreement, and association with future childbearing in northern Malawi. A total of 5,222 married women participated in the three-round study. The odds of having a child or becoming pregnant within 36 months were 4.2 times higher when both wife and husband wanted a child within three years and 2 times higher when both wanted to wait at least three years, compared with the odds when both wanted to cease childbearing. The influence of husbands’ and wives’ preferences on subsequent fertility was equal. Compared with the intention to stop, the intention to postpone childbearing was less stable, recorded less spousal agreement, and was much less strongly predictive of fertility.

The central aim of family planning programs is to meet couple’s unmet need for contraception and thereby reduce unintended pregnancies. Both of these outcomes are likely to be improved by timely survey information on reproductive preferences, which is typically elicited in three main ways: a question on total desired family size; prospective questions on whether another child is desired and, if so, when; and retrospective questions on whether recent births were wanted, mistimed, or unwanted at time of conception. Calculations of unmet need require data on both prospective and retrospective preferences together with contraceptive use (Bradley et al. 2012). Estimates of unwanted fertility can be made from any of the three types of preference data. Information on total desired family size is used to identify births in excess of desires, from which unwanted fertility rates can be generated (Westoff 2010). An alternative method of estimating unwanted fertility based on prospective information has been proposed by Casterline and El-Zeini (2007). The retrospective information provides direct estimates of unwanted (and mistimed) childbearing and, in conjunction with abortion data, has been used to determine levels and trends in unintended pregnancies (Singh, Sedgh, and Hussain 2010).

These three main methods of measuring unwanted births yield very different results. In a comparison of six Demographic and Health Surveys, the Casterline and El-Zeini method gave the highest proportions of births that were unwanted in all six surveys, and the retrospective method usually gave the lowest proportions. The reason for the differences is clarified by longitudinal studies. These invariably show that a large proportion of births to women who stated at baseline that they wanted no more children were retrospectively classified as wanted or mistimed (Westoff and Bankole 1998; Casterline, El-Zanaty, and El-Zeini 2003; Koenig, Acharya, and Singh 2006; Speizer et al. 2013; Jain, Mahmood, and Sather 2014). The usual interpretation of this discrepancy is ex post rationalization, but it is also likely to reflect a genuine difference between an abstract preference before an event, such as pregnancy, has occurred and a more emotional reaction to the event. Whatever the reasons, clearly no consensus exists on how best to obtain valid estimates of unwanted childbearing from DHS data, and this verdict also holds for the United States where the topic has attracted considerable attention from both social psychologists and demographers (e.g., Campbell and Mosher 2000; Santelli et al. 2003).

Doubts about the meaning of fertility preferences are most pronounced, and the relevant evidence is most sparse, in sub-Saharan Africa. Qualitative studies suggest that reproductive desires in this region may be unstable and tentative (Agadjanian 2005; Johnson-Hanks 2005), and prospective studies in Ghana and among young women in Malawi indicate a high degree of fluctuation in preferences over time (Kodzi, Casterline, and Aglobitse 2010; Sennott and Yeatman 2012; Yeatman, Sennott, and Culpepper 2013). In surveys in Burkina Faso, Ghana, and Kenya, between 25 and 40 percent of women who wanted to limit or postpone future childbearing reported that it would be no problem or a small problem if they became pregnant soon, indicating ambivalence about, or even indifference to, implementation of reproductive wishes (Speizer 2006). Whereas declines in fertility and in unmet need in other regions track each other closely, this is not true in sub-Saharan Africa, leading to further uncertainty about the meaning of preference data (Casterline and El-Zeini 2014).

To our knowledge, no comparison of prospective and retrospective preferences has been published from longitudinal studies in sub-Saharan Africa, and only two studies have examined the relationship between baseline desire for children and subsequent fertility. Bankole (1995) found that preferences in Nigeria were highly predictive of childbearing over a two-year follow-up. Among couples where both husband and wife wanted another child, 54 percent gave birth, compared with 8 percent where neither wanted another child and 23–25 percent where one spouse wanted a child and the other did not. In Ghana, the monthly odds of pregnancy were 5.3 times higher among women who wanted to become pregnant within two years than for women who wanted no more children (Kodzi, Johnson, and Casterline 2010). These results are in line with non-African studies, which also show that a stated desire to stop childbearing is strongly predictive of subsequent behavior.

In this article, we use longitudinal data from rural northern Malawi to assess the stability of childbearing desires over time, to compare prospective and retrospective classifications of intendedness of births and pregnancies, and to evaluate the predictive validity of both wives’ and husbands’ baseline childbearing preferences.

## Context

According to the 2010 Malawi Demographic and Health Survey (DHS), the total fertility rate in 2010 was 5.7 births per woman, a decline from 6.7 births in 1992. High fertility is accompanied by moderate use of contraception and a high level of unmet need. In the 2004 Malawi DHS, only 28 percent of women reported using a modern method of contraception, but this rose sharply to 42 percent in 2010. Despite this rise, one birth in every four in 2010 was reported to be unwanted. Between 2000 and 2010 the reported ideal family size decreased from 5 to 4 children per woman, and at the time of the 2010 survey 47 percent of married women said that they wanted no more children.

We use data from a Demographic Surveillance Site (DSS) established in 2002 in the Karonga district of northern Malawi. The DSS population numbered approximately 33,000 individuals and is fairly static; in-migration and out-migration rates are estimated as 72 and 98 per 1,000 person-years in 2008 (Crampin et al. 2012). Compared with other regions of Malawi, the northern region is the most rural but records the highest levels of education and literacy (Zulu 1996).

A range of contraceptives, including injectables, pills, and implants, along with tubal ligation, is provided by public and faith-based hospitals and health centers and by mobile services in the site. Women obtain injectables and pills mainly from hospitals or health centers (Dasgupta et al. 2013). While sterilization is extremely uncommon in sub-Saharan Africa, it has been popularized in Malawi in recent years through mobile clinics (Jacobstein 2013). As in other areas of the country, sterilization in Karonga is provided by Banja La Mtsogolo, a Marie Stopes affiliate. The level of contraceptive use in the DSS site is slightly higher than the national average. In 2008–09, 48 percent of married women reported current use, mainly hormonals (19 percent), condoms (19 percent), and sterilization (7 percent), with little difference according to HIV status (Dube et al. 2012). Use of a modern method was significantly higher among monogamous than polygamous couples but varied only modestly by future desire for children (Baschieri et al. 2013). Much less is known about abortion. Although the law is restrictive in Malawi, abortion incidence is estimated to be relatively high nationally at 23 per 1,000 women aged 15–44 years and is even higher (35 per 1,000) in the North (Levandowski et al. 2013).

Women’s median age at first marriage is 18, and the onset of childbearing is relatively early and universal with 90 percent of all 20–24-year-old women and 95 percent of currently married women of the same age having at least one child. The incidence of divorce and remarriage is high (Reniers 2003). The DSS community is patrilineal and residence after marriage is usually patrilocal (Peltzer 1987). This study notes that newly married couples are increasingly likely to live with neither the husband’s nor the wife’s relatives. Polygyny is a rooted social institution in this part of Malawi, with 15 percent of men and 27 percent of women in a polygynous relationship (Marston et al. 2009). The majority of polygynous couples (95 percent) live either in the same house or in the same compound, compared with 99 percent of monogamous couples. The average household size is around five members and the total fertility rate was 5.4 children per woman in 2009.

HIV prevalence in adults was estimated at 2 percent in the late 1980s and 13 percent in the late 1990s. Prevalence has been fairly stable since 2000, and the most recent study in 2009–10 estimated HIV prevalence at 9 percent in women and 7 percent in men (Floyd et al. 2012).

## Data and methods

The Fertility Intention Study (FIS) was conducted in the Karonga Prevention Study’s ongoing DSS site. FIS data were collected in three rounds, with an average interval of one year between rounds (Round 1: 28 October 2008 to 30 September 2009; Round 2: 1 October 2009 to 30 October 2010; Round 3: 1 November 2010 to 14 October 2011). All female and male residents in the DSS site aged 15–49 were eligible. All data presented here are restricted to currently married or cohabiting women and their partners.

The questions on women’s and men’s prospective and retrospective childbearing preferences were identical in meaning to those used in successive Malawi DHSs and were tested using the local language during the pilot study in 2008. The prospective measure ascertained desire for another child and preferred timing. Currently pregnant women were excluded from these questions. The questions were, “Do you want to have any (more) children any time in the future? (yes, no, or unsure)” and “If yes, how long would you like to wait before having another child?” The retrospective measure was obtained for all women who had a birth or reported a current pregnancy during any of the three rounds and ascertained whether the child or pregnancy had been wanted at that time, came earlier than desired, or had been unwanted. The question was, “At the time you became pregnant with your last child (born in the last three years) or in the current pregnancy, did you: a) want to become pregnant, b) want to wait until later, or c) want no (more) children at all?” These questions were asked in all three rounds. In addition, questions on marriage, fertility, and contraceptive use were asked. A total of 353 women were sterilized at first interview and were excluded from our study. However, the 231 women who were sterilized after the first interview were included. The women’s and husbands’ data were linked to create a matched couple file. Polygynous husbands were asked questions about their fertility intentions with each current wife.

The FIS followed the re-census of the site, which included the collection of vital events and individual and household socioeconomic status. The study module was nested in a module on sexual behavior and HIV sero-status. Consent for FIS participation was sought separately from consent for sexual and HIV testing in order to minimize refusal in the FIS component. This is an open-cohort longitudinal study, and immigrants to the site and women who became eligible to participate were enrolled in the later rounds.

The vital events and household migration of residents living in the site are recorded and updated in the Continuous Registration System (CRS). Because the individual records are linked with the mother and father’s identification number, live births in our analyses were identified by linking with this registration data. Pregnancy is self-reported in the FIS questionnaire; however, it is known that there is underreporting of pregnancy especially during the first trimester (Goldman and Westoff 1980). Thus, we classified a woman as pregnant at an interview if she gave birth within eight months following the interview. We had to rely on self-reported pregnancies and births to obtain the retrospective fertility preference measure because, self-evidently, this information can be asked only of women who report a birth or current pregnancy.

We included bivariate analysis to assess stability of fertility intention, comparability of prospective intention and retrospective fertility preference, and agreement of a couple’s fertility intention. We also estimated odds ratios of being pregnant or having at least one child within a three-year observation period using the matched couple data and logistic regression, which allows us to assess the effect of intentions after adjustment for demographic and social factors. All analyses were performed using Stata version 13.

## Results

A total of 5,222 nonsterilized married women participated in the fertility intention module at least once. Of these, 2,450 were interviewed in each of the three rounds, 1,602 were interviewed twice, and 1,170 were interviewed once (Table 1). In Round 2, 927 new women joined the study, and 426 women were interviewed only in Round 3. The response rates among eligible married women were 88 percent in Round 1, 91 percent in Round 2, and 83 percent in Round 3 (not shown). Among women who participated in all three rounds, 96 percent stayed married across rounds. The level of participation defines eligibility for specific analyses. For instance, examination of short-term stability of preferences requires participation in two consecutive rounds, while the matched couple analysis is based on couples who participated in Round 1.

The median age of all women who participated was 27 years, and the majority had at least two children. Nearly two-thirds had 6 to 8 years of primary schooling, and 22 percent had attended secondary school. Women who participated in all three rounds were slightly older and had more children than those who were interviewed once or twice (results not shown).

### Stability of Prospective Fertility Intentions

A total of 3,783 nonsterilized married women provided prospective fertility intentions in consecutive rounds. Among 6,233 pairs of observations derived from the 3,783 women who were interviewed in Round 1 and 2, and/or Round 2 and 3, 869 were pregnant at the first round, 29 did not provide prospective fertility intentions in the first round, and 25 did not provide the intention in the second round. In addition, the paired observations between Rounds 2 and 3 were excluded for women who underwent sterilization between Round 1 and 2, leaving 5,190 observations. At the first interview, 41 percent of women wanted no more children, 3 percent were unsure about having another child, 19 percent wanted to delay childbearing for three or more years, 4 percent wanted a child but were unsure about timing, and 33 percent wanted a child within three years. Examination of the stability of these intentions, or preferences, has to take account of changes in reproductive status. As shown in the last row in Table 2, 29 percent of the women became pregnant or gave birth between two consecutive rounds. Among women who wanted no more children in the earlier round, 62 percent gave the same response at the next round (or had become sterilized) (col. 1), 20 percent changed their response to wanting another child or were unsure (cols. 2 + 3 + 4 + 5), and 18 percent had given birth or were already pregnant (col. 6).

Stability is more difficult to assess for women who wish to postpone childbearing than for those who wanted to stop, because the passage of time will alter responses. Among women wishing to postpone the next child for at least three years, 27 percent had given birth or were pregnant at the next round (col. 6), and 12 percent changed their response to wanting no more children (col. 1). The remainder were divided between women who wanted a child within three years and those who still preferred to wait at least three years. Women who wanted a child but were unsure about the timing comprised 4 percent (190/5,190) of the sample. A third of these women were pregnant or had given birth by the subsequent interview (col. 6). One-fifth shifted to wanting no more children (col. 1), and another one-fifth changed their intention to wanting a child within three years (col. 5). Among women who stated at the earlier round a desire to have a child within three years, 23 percent gave an inconsistent response at the next round—almost equally divided between those who wanted no more and those who wanted to postpone for three or more years. The majority of women were already pregnant or gave the same response. Because the number of women who wanted a child but were unsure about the timing was small, and the proportion of women who became pregnant or had given birth between two consecutive interviews was similar to the proportion who wanted to have a child within three years, these women were combined with women who wanted a child within three years in subsequent analyses.

We also assessed the stability of prospective fertility intention between Rounds 1 and 3. Among those who wanted no more children at Round 1, 53 percent gave the same response at Round 3, 13 percent wanted another child or were unsure, while 35 percent were pregnant or had a child. Among women wanting another child at Round 1, a majority had a birth or pregnancy. Only about 5 percent reported that they now wanted no more children (results not shown).

### Comparison of Prospective Intention and Retrospective Attitude

Comparison of prospective and retrospective preferences requires information on prospective fertility intentions given before conception of the child and retrospective attitudes collected in a subsequent round after becoming pregnant or giving birth. This comparison is possible for 1,136 married women. For the 15 women who had more than one birth or pregnancy, data for the earlier event are used. In 319 cases (29 percent) the retrospective information was collected during pregnancy rather than after the birth. The retrospective preferences were similar in both groups (data not shown).

Under the prospective classification, unwanted births were defined as those occurring to women who wanted no more children. Mistimed births were defined as those occurring within 18 months to women who wished to wait for two to three years and those occurring within 30 months to those wishing to wait for three or more years. All other births or pregnancies, including those for which the woman was undecided about timing, were classified as wanted. The retrospective classification is based on the direct statements by the women.

Seventy-four percent of births defined as wanted prospectively were similarly defined retrospectively, as shown in Table 3 (col. 1). In contrast, only 14 percent classified as unwanted in the prospective measure were similarly classified retrospectively (col. 3). The majority of these births or pregnancies were reported as wanted or mistimed after they had occurred. Consistency of mistimed births was intermediate, with 41 percent agreement between prospective and retrospective measures (col. 2). Of the total 1,121 pregnancies or births, only 47 percent were classified consistently before and after the conception of the births. While the prospective measure showed 22 percent (250/1,121) of births or pregnancies to be unwanted and a further 38 percent (426/1,121) to be mistimed, the corresponding estimates from the retrospective measure were 4 percent unwanted (col. 3) and 32 percent (col. 2) mistimed.

### Perceived Consequences of Having a Child in the Next Year

Women who were not pregnant and wanted to delay the next birth for at least one year or to have no further children were asked, “If you have a child in the next year, will there be serious consequences? If yes, which consequences?” Table 4 shows the distributions of perceived consequences by fertility intention.

The proportion of women who reported that a birth would have serious consequences was highest among women who wanted no more children (69 percent). Around 30 percent of these women mentioned the pregnancy would have serious consequences for their own health, and the same proportion cited serious financial consequences. A majority of women who wanted to postpone the next child for three or more years also reported serious consequences (65 percent). Compared with women who wanted no more children, these women were less likely to mention financial matters but more likely to cite children’s health. Not surprisingly, women who wanted a child within three years or were undecided were less likely than others to report serious consequences of an early pregnancy.

### Husband’s Fertility Intention

To compare husbands’ and wives’ fertility preferences, we used matched couple data. Among 3,869 women who participated in Round 1, 2,244 women who were neither sterilized nor pregnant and provided prospective fertility intentions were matched with their husband’s data. Among them, 1,189 men were matched with one woman, 149 men were matched with two women, and 19 were matched with three women. The prevalence of polygyny in this subset of data (12.4 percent) is in line with the overall data, 16 percent of the husbands among the matched couples. A third of the men with multiple wives had different fertility intentions between the co-wives (data not shown). At the aggregate level, as shown in Table 5, 43 percent of wives (976/2,244) and 40 percent of husbands wanted no more children, and 17 percent of wives (382/2,244) and 13 percent of husbands wanted to postpone childbearing for three or more years.

At the individual level, as shown in Table 5, there is a high degree of agreement in the intention of couples to stop childbearing. Among wives wanting no more children, 67 percent of husbands gave the same answer (col. 1). Agreement between wives and husbands is at the same level for those who wanted to have a child within three years (col. 4). Postponement registers less spousal agreement. Among wives wishing to delay childbearing for at least three years, only 34 percent of husbands gave the same response, 22 percent wanted no more, and 39 percent wanted a child sooner than their wife or were unsure about the timing.

### Fertility Intentions and Behavior

We used multivariate logistic regression to further explore the predictive power of fertility intentions on reproductive behavior over a three-year time span. Because a preliminary analysis showed that the inclusion of the husband’s intentions improved the fit of the model (results not shown, likelihood ratio test: p-value <0.001), we used the subsample of matched couples. The covariates in the model were woman’s age, number of living children, education, and marriage type. Preliminary analysis showed that the addition of other covariates, such as household wealth and main source of income, had no effect on the results.

Model 1 in Table 6 shows that both wives’ and husbands’ fertility intentions predicted subsequent pregnancy or childbirth after adjustment for demographic and other factors. Compared with women who wanted no more children, the odds of pregnancy or birth for women who wanted another child within three years were 2.2 times higher and the odds for those who wanted to wait three or more years were 1.6 times higher. Examination of the 95 percent confidence intervals (not shown) indicates that the difference between those who wanted a child soon and those who wanted to delay is not significant. As indicated by the p-value, the difference between those who wanted a child soon and those who wanted to delay was not significantly different. The effect of the husband’s fertility intentions was strikingly similar to that of the wife.

One concern in Model 1 is the degree of collinearity between spousal preferences. Thus we ran a second model using joint preferences as a single variable (Model 2). The two models give similar results. Couples where both wanted a child within three years were 4.2 times more likely to experience pregnancy or birth within three years compared with those where neither of them wanted a child. The odds for couples where both wanted to wait three or more years and those where there was disagreement between spouses about desire for another child or the timing of an additional child were similar. Among other factors in the model, only the wife’s age was significantly related to childbirth or pregnancy.

The key result of Table 6 can be expressed in terms of the predicted probabilities of having a child or becoming pregnant by whether the wife, husband, both, or neither stated a desire to have more children in Round 1. When both spouses want another child, the predicted probability of a birth or pregnancy in the next three years is 0.63, holding other factors at their mean value. When only one spouse wanted no more children, the probability falls to 0.47 or 0.48, and it falls further to 0.33 when neither spouse wants any more children (results not shown).

Table 7 assesses whether a woman’s education and the intensity of her preference mediate the link between the desire to have no more children at baseline and subsequent childbearing. The odds of pregnancy or birth were about 30 percent lower for women who considered that an early pregnancy would have serious consequences for household finances or for her own or her children’s health, but this difference is of only borderline statistical significance. Contrary to expectations, education has no significant effect. Women’s age remains a very strong predictor: among women wanting to cease childbearing, the odds of pregnancy or birth are 80 percent lower for those aged 30 or older than for younger women.

## Discussion

We assessed prospective fertility preferences, or intentions, in terms of their temporal stability, intensity, degree of spousal agreement, and power to predict future childbearing. In similar studies conducted in Asia, the main interest has been on the distinction between those who want to stop childbearing altogether and those who want another child. In sub-Saharan Africa, those who want to postpone the next birth—an intermediate group—are of equal interest to those who want to stop. In this region, unmet need for family planning is more likely to arise from a desire to space births rather than to limit ultimate family size, although the proportion of women who want to cease childbearing has increased in many countries (Westoff 2012; Van Lith, Yahner, and Bakamjian 2013). Evidence suggests that fertility declines in sub-Saharan Africa are being driven by postponement, expressed in terms of very long birth intervals, rather than by the Asian and Latin American pattern of parity-specific cessation of childbearing (Timæus and Moultrie 2008; Moultrie, Sayi, and Timæus 2012).

Nevertheless, in this study population from rural northern Malawi, family-size limitation proved to be a more prevalent reported motive than postponement. Forty-one percent of women at the first interview said that they wanted no more children, a proportion close to the national estimate from the 2010 Malawi DHS, compared with 19 percent who wanted to delay the next child for three or more years. The desire to limit family size has been increasing in Malawi. For instance, in 1992 only 33 percent of women with four living children wanted no more children (or were sterilized). By 2010, this proportion had risen to 63 percent.

Some indicators in this study suggested that postponement was as compelling a motive as limitation for avoiding pregnancy. As shown by Baschieri et al. (2013), contraceptive prevalence in the two groups was similar and an equal proportion of women stated that serious consequences would ensue if they became pregnant within a year. However, a clear difference between limiters and postponers in the nature of these serious consequences was apparent. Compared with limiters, postponers were much less likely to mention financial consequences and much more likely to cite threats to children’s health. This difference is consistent with a large body of ethnographic evidence that the advantage of birth spacing for child health is widely understood in Africa.

Agreement between husband and wife about postponement was lower than agreement about limitation, and, most tellingly, the desire to have no more children was a much more powerful predictor of subsequent pregnancy or birth than the desire to postpone the next birth. Between Rounds 1 and 2—an average interval of 12 months—27 percent of postponers became pregnant or gave birth, compared with 18 percent of limiters and 45 percent of women who wanted a child within three years. After a lapse of three years, the corresponding figures were 55 percent for postponers and 63 percent among women who had wanted a child within this period, a difference that was not statistically significant. In contrast, 33 percent of limiters had given birth or were pregnant. Adjustment for women’s age and other possible influences on childbearing had little effect on estimates. It appears that postponement is an effective spur to avoid pregnancy in the short term but less so over a three-year period. This verdict is consistent with national trends in median birth-interval lengths, which have increased only modestly from 32.7 months in 1992 to 36.1 months in 2010, according to successive Malawi DHS reports.

The predictive power of baseline fertility intentions in this study is broadly in line with results from most other studies. In Morocco, 29 percent of women who wanted no more children gave birth or became pregnant within three years, compared with 62 percent of those who wanted another child (Westoff and Bankole 1998). In Egypt, the corresponding figures for a two-year observation period were 25 and 59 percent, and there was no difference between postponers and those who wanted a birth soon (Casterline, El-Zanaty, and El-Zeini 2003). Similarly, 34 percent of women in Pakistan who wanted no more children had a child or became pregnant within three years, compared with 68 percent of women who wanted another child; again no difference was observed between postponers and women who wanted a child soon (Jain, Mahmood, and Sathar 2014). Two studies in India gave contrasting results. In one study of three Indian states with a four-year follow-up, 51 percent of nonsterilized women who wanted no more children and 74 percent of those who wanted another child gave birth (Roy et al. 2008). In the other study, conducted in Uttar Pradesh, only 10 percent of nonsterilized women wanting to stop childbearing became pregnant or gave birth within two years, compared with about 50 percent of those who wanted to continue childbearing (Speizer et al. 2013).

Several factors may account for childbearing among women who state at baseline that they want no more children, including barriers to contraceptive adoption, discontinuation of use, accidental pregnancy while using a method, intentions that are weakly held or change over time, the influence of husbands having different views about childbearing, and the acquisition or loss of a co-wife by husband. In this study, nonuse of contraception among women who wish to cease childbearing is a major proximate cause of subsequent pregnancy or childbirth. At baseline, 48 percent of couples where both wanted no more children were not using contraception (Baschieri et al. 2013). Clearly, this population has a high unmet need for contraception. A greater emphasis on long-acting reversible methods, such as IUDs, might be effective.

Lack of intensity in the desire to avoid a future pregnancy provides a partial explanation of discrepancies between intentions and behavior. Thirty percent of women who desired no more children denied that any serious consequences would result from a birth in the next 12 months, a proportion similar to that reported in other African surveys (Speizer 2006), and these women were more likely to have a child than those who stated that a birth in the near future would have serious consequences. With regard to change in reproductive preferences, we were unable to determine its role directly, but evidence suggests that it may also have made an appreciable contribution. Among women stating a desire to have no more children at baseline and who were not pregnant at Round 2, 18 percent reported a desire to have another child. A similar proportion of women shifted from wanting another child within three years at Round 1 to wanting no more at Round 2. Thus there is a fair degree of short-term instability in preferences but not on a scale to undermine confidence in the meaning of responses. Moreover, changes are symmetrical.

One of the strengths of this study is the opportunity it gave us to assess the influence of husbands’ preferences on reproductive outcomes. We found a moderate agreement between spouses on desire for childbearing: 67 percent of husbands of women who wanted no more children gave the same response. The result is consistent with many previous studies showing similarity in aggregate in family-size preferences between husbands and wives, and with a recent study in which 83 percent of young Malawian couples had the same ideal family size or a difference of one child, and in which changes in desired family size among both men and women were strongly associated with partners’ preference, leading to spousal convergence (Yeatman and Sennott 2014). Nevertheless, 33 percent of matched couples in our study disagreed about future childbearing and/or its timing, and it was possible to compare the predictive power of husbands’ and wives’ intentions. The results were clear-cut: the intentions of both husband and wife matter and both are equally influential on the probability of future childbearing. The predicted proportions of having a child or becoming pregnant within a three-year period rise from 33 percent when spouses agree in their desire to stop childbearing to 47 or 48 percent when one wishes to stop but not the other and further to 63 percent when both want more children.

An extensive literature, reviewed by Blanc (2001), has examined the influence of both spouses’ preferences on contraceptive use from cross-sectional surveys, but very few prospective studies have collected relevant information independently from both partners. For Bangladesh, Gipson and Hindin (2009) found that the preferences of wives usually prevailed over those of husbands in cases of disagreement. The results from our study are more similar to the findings of Bankole and Singh (1998) in Nigeria, where the influence of husbands and wives was equal in terms of power to predict future childbearing. Interpretation should be cautious because wives may adapt their reproductive aspirations to the perceived wishes of their husband, as found in Malawi by Yeatman and Sennott (2014). However, both our study and Bankole’s challenge the often repeated claim that wives are relatively powerless to implement their desire to stop having children when their husband has different preferences.

In view of an earlier multivariate analysis that found contraceptive use to be higher among more-educated than among less-educated women and higher in monogamous than polygynous couples (Baschieri et al. 2013), it was also surprising that neither education nor marriage type modified the relationship between reproductive intentions and subsequent childbearing. Another finding of interest is that women’s age but not number of living children was a strong predictor of fertility. This is consistent with the expectation that fertility transition in Africa, in contrast to Asia, will be relatively even across age groups and will be less parity-specific (Caldwell, Orubuloye, and Caldwell 1992).

Our analysis has made a significant contribution to understanding the complexity of measuring unwanted childbearing. In the prospective assessment, 22 percent of births were classified as unwanted and a further 38 percent as mistimed. In contrast, only 4 percent of pregnancies or births were reported retrospectively as unwanted and 32 percent as mistimed. This contrast is consistent with other prospective studies and can only be partially attributed to changes in preferences. One puzzling feature is that the proportion of births classified retrospectively as unwanted in this study was much lower than in the 2010 Malawi DHS, despite closely similar wording of the question. An explanatory analysis showed that 27 percent of recent births to married women were reported as unwanted in the 2010 DHS, although this proportion was lower (18 percent) in Karonga district. Moreover, we found a high percentage of unwanted births among married women who had only one child (15 percent). This unexpectedly high estimate suggests that respondents to the DHS may have confused mistimed with unwanted childbearing, although it is also possible that retrospective estimates in our sample may be biased downward. Whatever the reason for the low estimate in this study, the contrast between prospective and retrospective estimates of unwanted childbearing supports the growing evidence that the prospective method invariably yields higher estimates than the retrospective method.

## Conclusion

The desire to limit family size has become much more common in Malawi over the past 20 years, but doubts have persisted about the interpretative weight that can be attached to such reproductive preferences in sub-Saharan Africa. The main conclusion of our inquiry is that the correspondence between the reported desire to cease childbearing and subsequent behavior in rural northern Malawi is similar to that found elsewhere, such as in Egypt, Morocco, and Pakistan. In other words, the predictive validity of the stated intention to stop childbearing found in this study is consistent with those observed in other regions. By contrast, the stated desire to postpone childbearing had a relatively weak predictive power over a three-year period.

The influence of the reproductive wishes of husband and wife on subsequent childbearing were symmetrical. While there was a fair degree of spousal agreement in whether or not to have another child, about one-third of matched couples expressed differing preferences. In these discordant cases, the probability of childbearing or pregnancy was appreciably higher than when spouses agreed to limit family size.

In line with other studies, we found unwanted childbearing to be much more common when measured prospectively than retrospectively. Our interpretation is that the two lines of questioning are estimating different constructs. The prospective questions tap a somewhat abstract desire for the future, while the retrospective questions elicit a more emotional reaction to an event that has already occurred. Given the high value traditionally attached to children in most African societies, it is not surprising that mothers are reluctant to define a child as unwanted.

## Figures and Tables

**Table 1:** Distribution of nonsterilized married women interviewed by rounds and eligibility for inclusion in specific analyses, northern Malawi

	Interview			Number of married respondents
Group	Round 1	Round 2	Round 3	
a	✓			424
b	✓	✓		726
c	✓	✓	✓	2,450
d	✓		✓	269
e		✓		320
f		✓	✓	607
g			✓	426
Total	3,869	4,103	3,752	5,222

NOTE: Table 2 includes women who were interviewed in consecutive rounds (Groups b, c, and f); Table 3 includes women who were interviewed at least twice (Groups b, c, d, and f); Tables 4–7 include women who participated in Round 1 (Groups a, b, c, and d).

**Table 2:** Stability of prospective fertility intention between two consecutive rounds (percent)

	Fertility intention at second round							
Fertility intention at first round	Wants no more children/became sterilized	Unsure about having a child	Wants to wait 3+years	Wants a child but unsure about timing	Wants a child within 3 years	Had birth or became pregnant between first and second round	Total	(N)
Wants no more children	62.4	2.2	6.2	2.2	9.3	17.6	100.0	(2,119)
Unsure about having a child	40.0	2.5	12.5	5.6	18.8	20.6	100.0	(160)
Wants to wait 3+ years	12.4	1.7	28.5	2.8	27.1	27.4	100.0	(1,006)
Wants a child but unsure about timing	20.5	2.6	14.2	8.4	21.6	32.6	100.0	(190)
Wants a child within 3 years	8.3	1.0	10.6	3.1	31.7	45.3	100.0	(1,715)
Total	32.6	1.7	12.5	2.9	20.9	29.3	100.0	(5,190)

**Table 3:** Comparison of prospective fertility intention and retrospective attitude (percent)

	Retrospective measure					
Prospective measure	Wanted to become pregnant	Wanted to have later	Wanted no (more)	Unknown	Total	(N)
Wanted/wanted but unsure about timing	74.4	20.2	1.6	3.8	100.0	(425)
Mistimed	56.3	40.6	0.7	2.3	100.0	(426)
Unwanted	46.0	37.6	13.6	2.8	100.0	(250)
Unsure about having a child	50.0	40.0	5.0	5.0	100.0	(20)
Total	60.7	32.2	4.0	3.0	100.0	(1,121)

**Table 4:** Perceived consequences of having a child in the next year by fertility intention at Round 1 (percent)

	Wife’s fertility intention				
Type of serious consequence	Wants no more children	Unsure about having a child	Wants to wait 3+years	Wants a child within 3 years/unsure about timing	Total
Household finances	28.0	16.3	9.9	5.8	16.8
For one’s own health	29.0	16.3	27.4	17.7	24.5
For children’s health	4.5	6.7	25.4	25.6	15.5
Other	7.3	5.9	2.7	3.1	5.0
None	30.4	52.6	32.8	46.2	36.9
Missing	0.8	2.2	1.8	1.5	1.3
Total	100.0	100.0	100.0	100.0	100.0
(N)	(1,319)	(136)	(601)	(971)	(3,027)

NOTE: 281 women who wanted a child within a year were not asked this question.

**Table 5:** Comparison of wife’s and husband’s fertility intentions in Round 1 (percent)

	Husband’s fertility intention						
Wife’s fertility intention	Wants no more children	Unsure about having a child	Wants to wait 3+ years	Wants a child within 3 years/unsure about timing	No intention given	Total	(N)
Wants no more children	66.6	3.2	6.2	17.9	6.1	100.0	(976)
Unsure about having a child	46.1	11.2	6.7	31.5	4.5	100.0	(797)
Wants to wait 3+ years	21.7	3.9	33.5	38.5	2.4	100.0	(382)
Wants a child within 3 years/unsure about timing	15.3	2.0	12.9	66.9	2.9	100.0	(89)
Total	39.9	3.2	13.3	39.4	4.2	100.0	(2,244)

**Table 6:** Adjusted odds ratios for childbirth or pregnancy within three years of Round 1 interview

	Odds ratio	
	Model 1	Model 2
Wife’s fertility intention		
Wants no more children	1.00	
Unsure about having a child	1.30	
Wants to wait 3+ years	1.59**	
Wants a child within 3 years/unsure about timing	2.24***	
Husband’s fertility intention		
Wants no more children	1.00	
Unsure about having a child	1.72*	
Wants to wait 3+ years	1.55**	
Wants a child within 3 years/unsure about timing	2.02***	
Missing	1.26	
Joint fertility intention		
Both want no more children		1.00
Both or one spouse unsure about having a child, or no husband intention		1.99***
Both want to wait 3+ years		2.07**
Husband wants more or sooner than wife		2.51***
Wife wants more or sooner than husband		2.74***
Both want a child within 3 years		4.21***
Wife’s age		
15–29	1.00	1.00
30–49	0.34***	0.33***
Number of living children		
0–3	1.00	1.00
3–4	1.07	0.99
5+	1.29	1.17
Type of marriage		
Monogamous	1.00	1.00
Polygynous	0.99	0.97
Wife’s educational status		
None/primary 1–5 years	1.00	1.00
Primary 6–7 years	1.27	1.28
Primary 8 years	1.19	1.20
Secondary+	0.91	0.91
(N)	(2,063)	(2,063)

* Significant at p < 0.05; **p < 0.01; ***p < 0.001.

**Table 7:** Adjusted odds ratio for childbirth or pregnancy within three years of Round 1 interview among married women who wanted no more children

	Odds ratio
Woman’s intensity of unwantedness	
No or nonspecified serious consequence	1.00
Serious consequence for household finances or for own or children’s health	0.72*
Husband’s fertility intention	
Wants no more children	1.00
Unsure about having a child	1.69
Wants to wait 3+ years	1.59
Wants a child within 3 years/unsure about timing	2.09***
Missing	1.76
Wife’s age	
15–29	1.00
30–49	0.21***
Number of living children	
0–3	1.00
3–4	0.92
5+	1.29
Type of marriage	
Monogamous	1.00
Polygynous	1.20
Wife’s educational status
None/primary 1–5 years	1.00
Primary 6–7 years	1.58
Primary 8 years	1.47
Secondary+	0.96
(N)	(796)

* Significant at p < 0.05; **p < 0.01; ***p < 0.001.
